# Impact of the COVID-19 pandemic on the care of rare and undiagnosed diseases patients in France: a longitudinal population-based study

**DOI:** 10.1186/s13023-022-02580-7

**Published:** 2022-12-09

**Authors:** Louis Soussand, Mathieu Kuchenbuch, Claude Messiaen, Arnaud Sandrin, Anne-Sophie Jannot, Rima Nabbout

**Affiliations:** 1 Banque Nationale de Données Maladies Rares, DSI-Innovation and Données, APHP, Paris, France; 2 Department of Pediatric Neurology, Reference Centre for Rare Epilepsies, Hôpital Necker-Enfants Malades, 149 Rue de Sèvres, 75015 Paris, France; 3 Laboratory of Translational Research for Neurological Disorders, INSERM-MR1163, Imagine Institute, Paris, France; 4 AP-HP. Centre - Université de Paris Cité, Paris, France; 5 HeKA Centre de Recherche des Cordeliers Inserm, INRIA, Paris, France

**Keywords:** Rare diseases, COVID-19, Health policy, National health registry, National health program, Telehealth

## Abstract

**Background:**

Preliminary data suggest that COVID-19 pandemic has generated a switch from face-to-face to remote care for individuals with chronic diseases. However, few data are available for rare and undiagnosed diseases (RUDs). We aimed to assess the impact of the COVID-19 pandemic on the activities of the French reference network for RUDs in 2020.

**Results:**

In this longitudinal retrospective study, we extracted and analyzed the data of the French national registry for RUDs collected between Jan 1, 2019 and Dec 31, 2020. We compared the annual longitudinal evolution of face-to-face and remote care activities between 2019 and 2020 focusing on adult and pediatric patients. Compared to 2019, rare diseases (RD) care activities showed a decrease in 2020 (− 12%) which occurred mostly during the first lockdown (− 45%) but did not catch up completely. This decrease was mainly in face-to-face care activities. Telehealth activities showed a 9-fold increase during the first lockdown and was able to cover for one third of the decrease in RD activities. Finally, the total number of patients receiving care was lower in 2020(− 9%) with a drastic decrease of cases with newly confirmed diagnosis (− 47%).

**Conclusion:**

Although telehealth was quickly introduced during the COVID-19 pandemic, RUD patient care was strongly affected in France with a decline in the number of patients treated and new patients recruited. This is likely to result in delays in patient diagnosis and care over the next few years.

**Supplementary Information:**

The online version contains supplementary material available at 10.1186/s13023-022-02580-7.

## Background

France was the ninth most impacted country by the COVID-19 pandemic in terms of confirmed cases per million people between 22 January and 31 December 2020 [1]. Like several countries facing the coronavirus outbreak, France’s government triggered nationwide lockdowns to limit the spread of the virus. In 2020, French population had two lockdowns: from 17 March to 11 May 2020 and from 30 October to 1st December 2020. These periods were associated with restricted access to health facilities giving priority to emergencies and COVID-19 patients.

There are 6100 diseases identified as rare and undiagnosed diseases (RUDs) according to the online database on rare diseases Orphanet [2–5], i.e., disease with a prevalence less than 1 in 2000. The COVID-19 pandemic has had many impacts on people with RUDs, including on health status, daily life, social life, financial status and mental health [6, 7]. People with RUD are particularly vulnerable to pandemics such as COVID-19. This is due on one hand to structural factors, such as small numbers of specialized centers with multidisciplinary and high expertise [8] and on the other hand to individuals with RUDs factors as they usually have with multiple co-morbidities, some of which favor the development of severe forms of COVID-19 in addition to phobia and anxiety [7]. This has led many patients with RUDs to avoid coming to the hospital as much as possible for fear of contracting COVID-19 [7–9]. The COVID-19 pandemic has thus led to the cancellation or postponement of large numbers of follow-up or first visits and even of some treatment’s procedures. The hospitals teams and activities submerged by the needs of the patients with COVID-19 and the fear of the RUDs patients lead to a cancellation magnitude not encountered previously [6, 8, 10]. To cope with these difficulties, many health institutions have switched from face-to-face encounters to telehealth activities [11, 12]. However, there are no quantitative assessments of these organizational changes neither of their variability after the lockdown at national scale.

In this study, we aimed to use the French national RUDs data registry, an unprecedented support for epidemiological, clinical and therapeutic studies in the field of RUDs [13, 14], to quantify longitudinally the impact of COVID-19 pandemic throughout the year 2020 and to compare it to 2019 as a reference year. We studied the temporal trends of face-to-face and remote activities in relation to French health policies during COVID-19 pandemic, especially lockdowns.

## Methods

### Study design

In 2004, France launched a national plan for rare diseases to improve patients’ care for individuals with RUDs. This plan led to the labelling of 131 reference centers, often multicentric, dedicated to a specific rare disease, such as cystic fibrosis, or a group of similar rare diseases, such as “rare epilepsies”, or “neuromuscular diseases”. These centres were further grouped in 23 specialty networks as “Brain team” for rare brain disorders. These reference centers cover the entire French territory and include 2114 sites or health institutions. In 2017, a nationwide initiative launched with the rare diseases data registry (BNDMR) aiming to include all rare disease sites activities in France [13] and to collect a common set of data shared by all rare diseases [15].

We conducted a longitudinal retrospective study based on the BNDMR population-based cohort to address the changes in the main clinical activities for patients with RUDs. We included patients’ visits in 2019 and 2020 corresponding to day and conventional hospitalization and face-to-face or remote encounters. This study was assessed by the scientific committee of the BNDMR as requested by data governance authorized by the French Data Protection Authority (Authorization DR-2019-113).

### Statistical analysis

We first excluded data from sites without comparable activity trend between January/February 2019 and January/February 2020, both periods being before COVID-19 pandemics. This was necessary because of the progressive deployment of the software application provisioning data into the BNDMR data base. First, we discarded sites with no activity at any given month of 2019 or 2020. Then, we used the isolation forest method [16] to detect sites with abnormal behavior regarding the proportion of activities in January/February 2019 over the entire year of 2019, the proportions of activities in January/February 2020 over the entire year of 2020 and in January/February 2019 and January/February 2020. We provide median with interquartiles for all the variables of interest (descriptive statistics). We used the non-parametric Wilcoxon tests to identify differences in distributions of sites activity between 2019 and 2020. First, we compared the distribution of activities by sites. Second, we investigated the difference between adult and child patients’ activities by evaluating the difference in the number of activities by site on adult patients only and child patients. The threshold for adult patient was determined according to the majority age in France (18 years) calculated on December 31th 2020. Third, we compared the differences in distribution of sites’ activities between the lockdown and non-lockdown periods of 2019 and 2020. Finally, we compared the activities by site during the different periods of the year using Wilcoxon test on the following periods: pre-COVID-19 pandemics period (from January 1st to March 17th and from September 1th to October 14th), first lockdown (from March 17th to May 11th), deconfinement (from May 11th to July, 4th), summer break (from July, 4th to September 1th), second lockdown (from October 14th to December 15th) and Christmas break (from December 15th to December 31th). As we perform a test for each period and age group (child/adult), i.e., 9 independent tests, we considered for this secondary analysis a significance threshold of 0.5%, which corresponds to the significance threshold using a Bonferroni correction [17].

We repeated the same analyses for face-to-face encounters on one hand and telehealth activities on the other hand. For this secondary analysis, we also considered multiple testing and set the threshold of significance to 0.06% (9 tests for each type of activity). We calculated the distance between patients’ home and care site using Haversine distance which computes the smallest distance between two points on a sphere [18]. The analysis was performed using R version 4.0.3 [19]. A *p*-value < 0.05 was statistically significant, and a *p*-value < 0.1 was a tendency.

## Results

### Characteristics of the population and evolution of overall activities during 2020

**Fig. 1:**
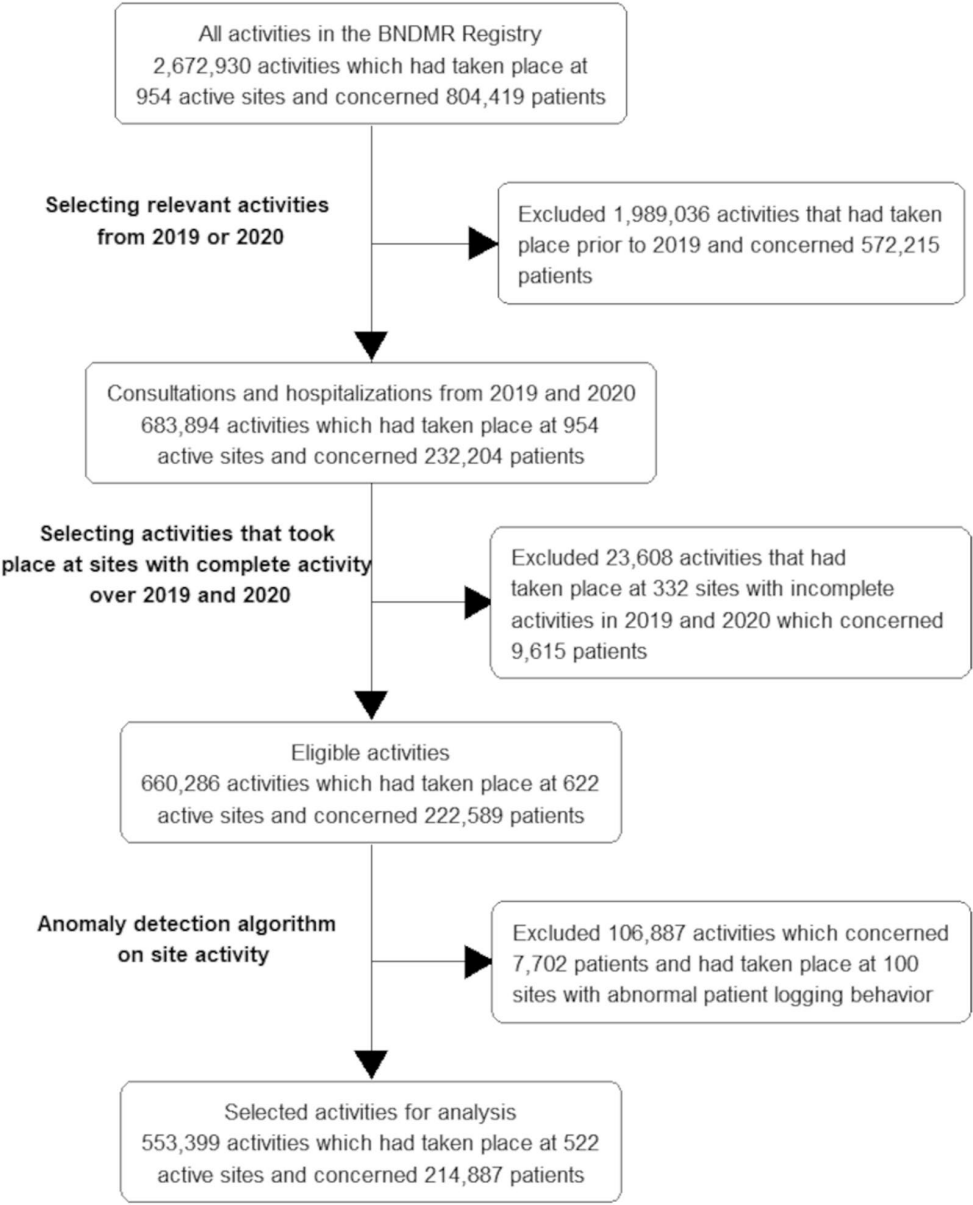
Flow diagram for the selection of eligible sites for this study

We included 209,403 from the total 232,204 patients for whom at least one activity was recorded during 2019–2020 in the BNDMR. Patients were included in 522 RUDs reference sites and generated 563,399 activities (Fig. 1). Table 1 summarized the characteristics of the population. The number of patients having an activity recorded decreased from 145,056 in 2019 to 132,282 in 2020, the year of the COVID-19 pandemic (− 9%). The number of patients with a newly confirmed diagnosis decreased from 16,799 in 2019 to 8949 in 2020 (− 47%). There was a 13% decrease from 300,019 activities in 2019 to 263,380 activities in 2020. Activities per site were not statistically different for the entire cohort and for the pediatric population but there was an almost statistical tendency for the adult population activities that decreased from 175,431 in 2019 to 143,654 in 2020 (101 [22–383.5] activities per site in 2019 to 87 [18–298.5] activities per site in 2020, *p* = 0.14). The number of activities per site significantly decreased during lockdowns periods from 64 [15–189] in 2019 to 23 [8–82] in 2020, yet the decrease outside of the lockdown periods was not statistically significant. When looking into the difference of activity between the periods, the first lockdown was the only part of the year that showed statistical significance from 46,019 activities in 2019 to 25,444 activities in 2020 (33 [8–105.25] activities per site in 2019 versus 19 [6–59] activities per site in 2020, *p* < 0.001) while the other periods did not show significant difference between 2019 and 2020 (Additional file 1: Table S1A). More precisely, his decrease mostly involves face-to-face activities. Face to face encounters showed a decrease from 65% of the whole care activities in 2019 to 59% in 2020 while hospitalization showed a lighter decrease (respectively from 16 to 14% for day hospitals and from 8.5 to 7.5% for conventional hospitalization (Additional file 1: Table S2). However, telehealth activities significantly increased in 2020 by 277.1% (n = 27,846, i.e., from 2.5% in 2019 to 10.6% of the whole care activities in 2020). However, specialty networks changes were heterogeneous. For instance, there was only a small difference in care activities between 2019 and 2020 for the network specialized in somatic or cognitive developmental anomalies (+ 2.5%) as well as for the rare and undiagnosed autoimmune and auto-inflammatory diseases (− 1%) in contrast to a major drop for the rare endocrine diseases (− 33%) and the neuromuscular diseases networks (− 22%), (see Additional file 1: Table S3).

**Table 1:** Characteristics of the individuals with rare and unknown disease of the 522 RUDs reference sites involved in this study between 2019 and 2020

	All		Children		Adults	
	2019	2020	2019	2020	2019	2020
Patients (n)	145,056	132,282	58,087	56,124	85,076	73,839
Sex ratio (M/F)	0.98	0.99	1.17	1.17	0.86	0.87
Number of visits (n)	300,019	263,380	122,155	116,347	175,295	143,565
Age (years)	25 [10–55]	22 [9–52]	8 [4–13]	8 [3–12]	50 [32–66]	49 [32–65]
New confirmed diagnosis (n)	16,799	8949	8306	5113	8494	3837
*Levels of Diagnostics (n)*						
Confirmed	223,012	188,304	82,748	75,562	139,513	111,578
Ongoing confirmation	34,104	36,468	16,294	19,398	16,897	15,696
Probable	20,065	19,910	9110	10,000	10,699	9624
Unknown	19,830	15,623	12,438	9821	6755	5177
Missing	3008	3075	1565	1566	1431	1490

### Focus on face-to-face care activities evolution during 2020

**Fig. 2:**
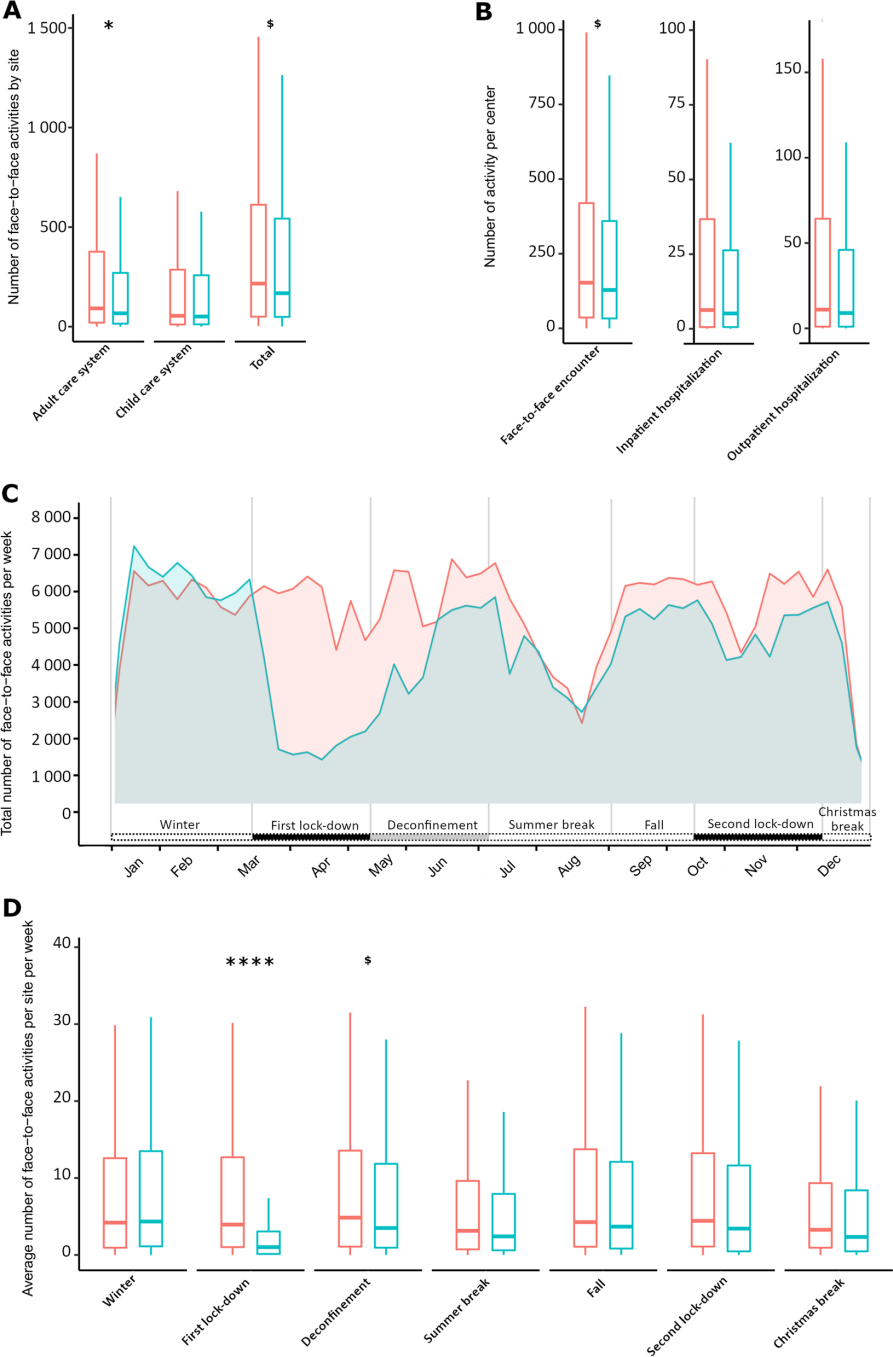
Impact of COVID-19 pandemic on face-to-face care activities in 2020 and comparison with the same activities in 2019 in 522 RUDs reference sites. **A**. Total face-to-face care activities in 2019 (in red) and in 2020 (in black) in the paediatric and adult care. **B**. Evolution of the different face-to-face activities between 2019 and 2020, **C**. Evolution of the total number of face-to-face activities per week in 2020 (in blue) according to the health policies implemented to face COVID-19 pandemic (in the lower part), the number of deaths in 2020 due to COVID-19 pandemic per day (in black) and the face to face activities in the same centres in 2019 (in red). **D**. Comparison of face-to-face activities between 2019 and 2020 by centre according to the different periods of the health policies implemented in France during COVID-19 pandemic. ****p < 10^–4^, ***p < 0.001, **p < 0.01, *p < 0.05, ^$^*p* < 0.1

The year 2020 was associated with a 20% decrease in the number of face-to-face activities between 2019 and 2020, from 292,634 to 235,534. There was a statistical tendency of the number of activities per site between 2020 and 2019 (with 217.5 [51–638.75] face-to-face activities per site in 2019 compared to 170 [50–545.25] per site in 2020, *p* = 0.09). This decrease in face-to-face care appears to have affected mostly the adult population (100.5 [22–384.5] per site in 2019 versus 72 [16–284.5] in 2020 for adults’ care site, *p* = 0.03) but seem to have had a limited impact on the pediatric population (64 [13–304] activities per sites in 2019 versus 54 [12.75–259] in 2020 activities per children’s care site, *p* = ns, Fig. 2A). Concerning the type of activities, all face-to-face activities but more particularly the regular consultations were the most negatively impacted. They dropped from 194,328 in 2019 to 154,348 (143 [32–417] activities per site in 2019 versus 115 [28–319] in 2020, *p* = 0.06) followed by the day hospitalizations with 48,217 in 2019 to 38,520 (23 [5–90] activities per site in 2019 versus 17 [4–71] in 2020, *p* = 0.14), (see Fig. 2B and Additional file 1: Table S2).

The number of activities decreased significantly during the lockdowns from 85,995 in 2019 to 33,987 in 2020 (per site: 64 [15–179] in 2019 to 16 [6–51], *p* < 0.001) while it was not statistically different during the non-lockdown periods of the year. For the face-to-face encounters, there were statistically significant differences between 2019 and 2020 during the first lockdown (33 [8–103] in 2019 and 10 [4–31] in 2020, *p* < 10^–4^) and a statistical tendency during the end of lockdown period (37 [9–109] in 2019 and 39 [8–93] in 2020, *p* = 0.09) but other periods of the year were not significantly different (Additional file 1: Table S1B, Fig. 2C, D).

### Focus on the development of telehealth during 2020

**Fig. 3:**
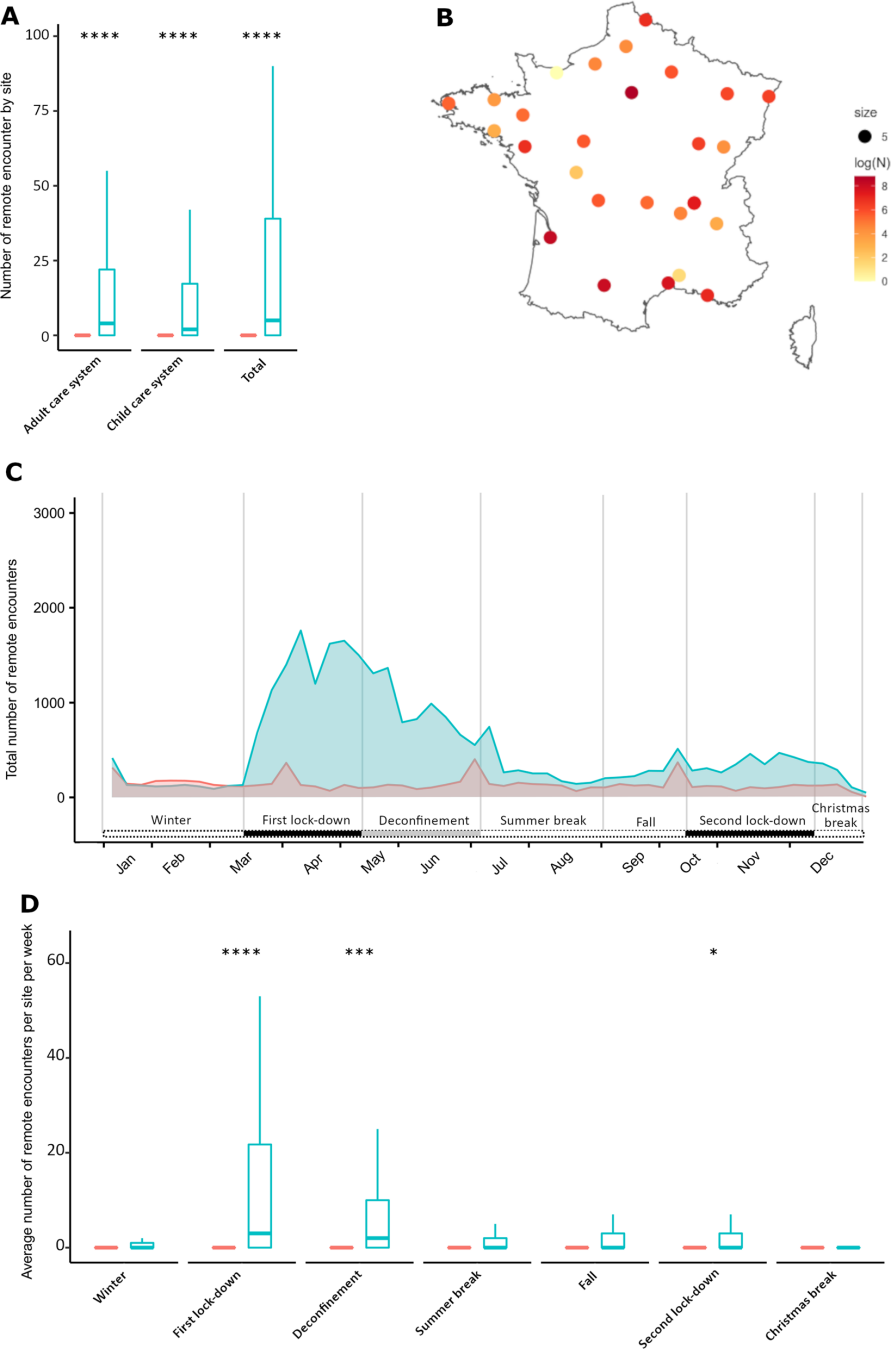
Impact of COVID-19 pandemic on telehealth in 2020 and comparison with 2019 in 522 RUDs reference sites. **A**. Total telehealth activities in 2019 (in red) and in 2020 (in black) in paediatric and adult populations, **B**. Location of the telehealth activities developed in the different reference centres for RUDs throughout France, **C**. Evolution of the total number of telehealth activities per week in the year 2020 (in blue) according to the health policies implemented to face COVID-19 pandemic (in the lower part), the number of deaths in the year 2020 due to COVID-19 pandemic per day (in black) and telehealth activities in the same sites in 2019 (in red). **D**. Comparison of telehealth activities between 2019 and 2020 by centre according to the different periods of the health policies implemented in France during COVID-19 pandemic. ****p < 10^–4^, ***p < 0.001, **p < 0.01, *p < 0.05, ^$^*p* < 0.1

The year 2020 was marked by the emergence and development of telehealth (27,846 activities versus 7385 in 2019, + 277% increase). Indeed, in 2019, only 50 sites provided this type of remote encounters (9.6%) compared to 359 (68.8%) in 2020. The number of telehealth activities thus increased from 2 [1–14] per year per site in 2019 to 19 [4–72] in 2020 (*p* < 10^–4^, Fig. 2A). This increase was heterogeneous as it was mostly developed in reference sites in large cities (over 200,000 inhabitants). Indeed, these eleven French cities alone account for 86.9% of the telehealth activities during COVID-19 pandemic (n = 24,224 encounters, Fig. 3B). The number of telehealth activity rose significantly for adult patients, from 3194 in 2019 to 15,812 in 2020 (2 [1–11.25] activities per site in 2019 versus 12 [3–43] in 2020, *p* < 10^–4^) and pediatric patients, from 4004 activities in 2019 to 11,640 in 2020 (2 [1–14.25] activities per site in 2019 versus 9 [2–44.5] in 2020, *p* < 10^–4^) (Fig. 3A). The teleconsultation activities increased significantly between 2019 and 2020 during the first lockdown, from 1165 in 2019 to 11,527 in 2020 (3 [1–12] activities per site in 2019 versus 13 [3–38.75] in 2020, *p* < 10^–4^), and a tendency remains at the end of the lockdown, from 1253 in 2019 to 6935 in 2020 (2 [1–16] activities per site in 2019 versus |7 [3–26] in 2020, *p* = 0.002), and at the second lockdown from 720 to 2627 (2 [1–14.5] activities per site in 2019 versus 4 [2–14] in 2020, *p* = 0.03) (Fig. 3) (Fig. 3C, D). The number of telehealth activities per site were not significantly different during the other periods (Additional file 1: Table S1C). This shift in the number of telehealth activities nationwide between 2020 and 2019 has reduced patient travel. We estimated that telehealth activities in 2019 saved 1,544,234 km of patients’ travels, equivalent to 39 times around the earth, *versus* 6,199,610 km in 2020, 155 times around the earth (+ 400% of saved kilometers in 2020 compared to 2019).

## Discussion

While the COVID-19 pandemic has had an impact on care networks, little is known about the impact of COVID-19 pandemic on RUDs activities in a national-health system [20]. Our study confirmed that the COVID-19 pandemic had a strong impact on the management of non-COVID affected individuals with rare diseases.

Using a national RUDs registry (BNMDR), we identified a decline in face-to-face activity, starting in March 2020 and continuing through the year. This decline in face-to-face activities was striking during the first lockdown (− 69%). During the same period, there was a strong increase in telehealth activities (+ 890%) that continued during 2020 (+ 277.1% for the whole French rare disease network), mainly during the first lockdown and the in the period following its end. This telehealth activity development filled 35% of the decrease in activities but did not allow to catch-up in the number of patients included in the RUD care system in 2020 when public health conditions improved. Thus, the French RUD networks have recorded a decrease in the total number of first and follow-up activities (− 12%) and a decrease in the number of individuals followed (− 9%) in 2020.

The impact of the COVID-19 pandemic as a break in the continuum of care was well identified in many chronic diseases. On one hand, in response to the pandemic, many countries had to reallocate resources from chronic pathologies to the care of individuals suffering from COVID-19. On the other hand, due to the fear of COVID-19 contamination, a number of individuals with rare diseases postponed their medical follow-ups [6]. In the Asia–Pacific region, 89% of rare disease health institutions and organizations have been affected by the COVID-19 pandemic, 63% have had their capacity decreased and 42% have had their funding reduced [10]. In Hong-Kong, 71% of individuals with RUDs reported a reduction in clinical visits [6]. In our study, we found a striking impact of the first lockdown on the number of encounters in the French rare disease network (− 74% per rare disease centre). These data are consistent with those of the Campania Rare Disease Registry, which show a 77% decrease in the number of new rare disease diagnosis in March–April 2021 compared to the same period in 2019 [21]. During this period, many caregivers and practitioners have been reassigned in clinics to favor the management of patients with COVID-19. This might partly explains the decrease in diagnosis and management in RUDs networks. However, it is important to note that this delay linked to the first-lockdown period did not catch up during the rest of the year 2020. Indeed, the number of encounters in the rest of the year remained below that of 2019. Adult patients, especially new patients, seemed to be the most affected in this decrease of encounters.

The impact of COVID-19 pandemic on the management of non-COVID-19 patients is well documented in the oncology field [22]. A decrease in the number of new cancer diagnoses is highlighted. This is notably due to the temporary suspension of screening campaigns, a decrease in the number of visits to the general practitioner and an increase in the time taken to carry-out expert clinical, laboratory and imaging investigations [23, 24]. For instance, in United Kingdom, the rate of skin cancer diagnosis decreased by 68% between March and June 2020 [24]. The number of individuals waiting more than 6 weeks for investigations (Computed Tomography scan, endoscopies, Magnetic Resonance Imaging, ultrasonography) increased tenfold in August 2020 compared to the same period in 2019 [24]. This delay in the management will lead to more patients with late-stage diagnosis and ultimately to increased mortality. Thus, the excess mortality from colorectal and lung cancers is estimated to increase till 10,000 deaths over the next 10 years in the United States [25]. COVID-19 pandemic has also modified the care trajectories to adapt to the structural impact of COVID-19. The lack of intensive care beds occupied by COVID-19 patients caused a delay in surgeries and patients who needed cancer surgery as first line therapy received radiotherapy as an alternative therapy waiting for the surgery availability.

On the same line, the frequency of chemotherapy and/or radiotherapy sessions was arbitrarily decreased [25]. Another important effect of the COVID-19 pandemic is the suspension of the clinical trials or the deviation from the previewed protocols [24, 26]. For example, the clinicaltrials.gov website reported 905 clinical trial suspensions due to COVID-19 pandemic from a total of 1052 studies between March and April 2020 [26]. It is currently difficult to quantify the impact of such changes on the long-term outcome especially for RUD population. However, 46% of people with RUD in the Hong Kong study estimated a decline in their health status [6]. Moreover, patients claimed that COVID-19 pandemic, especially because of the limited access to care and sometimes to treatment, has affected their mental condition [6, 27–29].

To face these difficulties, national and local guidance have been urgently provided to develop and facilitate telehealth [30]. Some health care systems have swapped all or part of their encounters for remote ones [9, 11, 12, 31]. Many studies on telehealth have asked the question: what will be the evolution of this type of encounters after the COVID-19 pandemic [9, 11, 12]? Our study, like others in primary care, shows a strong increase in this practice during the first lockdown (+ 890%) [31]]. During the rest of 2020, we recorded an increase of the number of telehealth activities compared to 2019. Indeed, most sites began to offer telehealth due to the pandemic (9.6% in 2019 versus 68.8% in 2020). However, the evolution of this type of encounter seems to be opposed to face-to-face encounters. We have thus progressively identified a decrease in the number of telehealth activities in parallel with a progressive reintroduction of face-to-face encounters. In a study to assess caregivers’ and physicians’ satisfaction with telehealth, we found that only 19.6% of practitioners agreed to do a second encounter using telehealth tools after a first remote encounter, expressing the need to meet physically their patients [27]. Although there has been an increase in telehealth activities, the number of total activities in 2020 failed to reach the numbers of 2019 numbers. We hypothesized that human resources are the limiting factor. This explains why there has been no real catching up in the number of cases after the periods of lockdown.

In this study, we acknowledge some limitations. This is a retrospective study based on a database that has new sites opening regularly. However, this should have increased the numbers and activities and patients in 2020 and should not bias the decrease. We could not explore the causes of the individuals’ loss of follow-up. This would have allowed us to better identify the means to deal with this loss of follow-up in other similar situations. The approach to the study relied heavily on selecting comparable sites in term of data entry, which creates by design several biases in the analysis. First, we eliminated sites with incomplete data over the year thus removing sites that had only recently joined the system. Second, we used an anomaly detection method to select sites based on the congruence of its activities during pre-pandemic periods to identify comparable sites. This approach permitted to study the impact of theCOVID-19 pandemic on reliable sites with years of logging activity and compare 2019 and 2020 but it kept out the new sites that joined the initiative during that time. It is important to note that the impact of the COVID-19 pandemic has not been the same in different countries, as shown by the study of different national organ transplant registries [32]. An international study could be useful to compare and study those variable factors and their effects on the impact of the pandemic.

## Conclusions

Although the healthcare networks were able to adapt quickly to COVID-19 situation by innovating, in particular by developing telehealth, it is important to note that this adaptation only limited the damage caused by the lockdowns and the resulting limitation of face-to-face care. These results should push healthcare networks to develop innovative solutions in case of health crises such as the COVID-19 pandemic. In addition, healthcare networks should be aware of the possible long-term impact of such pandemic. Tracking patients lost to follow-up and potential new patients who failed to reach specialized centers for rare diseases is today priority to prevent them from being left behind and to limit delays in diagnosis and care.

## Supplementary Information

**Additional file 1**: **Table S1**. Wilcoxon Tests to compare 2019 and 2020 activities in the BNDMR cohort. **Table S2**. Wilcoxon Tests to compare 2019 and 2020 activities by context in the BNDMR cohort. **Table S3**. Numbers of activities by specialty network in France.

## Data Availability

The datasets used and/or analysed during the current study are available from the corresponding author on reasonable request.

## Abbreviations

RUDs: Rare and undiagnosed diseases
BNDMR: French rare diseases data registry
