# Supplementary material for: Impact of the COVID-19 pandemic on the care of rare and undiagnosed diseases patients in France: a longitudinal population-based study
Source: Orphanet J Rare Dis. 2022 Dec 9;17:430. doi: 10.1186/s13023-022-02580-7 (PMC9733228; doi:10.1186/s13023-022-02580-7)
Supplement: Supplementary file 1 — Additional file 1: Table S1. Wilcoxon Tests to compare 2019 and 2020 activities in the BNDMR cohort. Table S2. Wilcoxon Tests to compare 2019 and 2020 activities by context in the BNDMR cohort. Table S3. Numbers of activities by specialty network in France. [file 13023_2022_2580_MOESM1_ESM.docx]

|  | | | Total 2019  N | | Total 2020  N | | Total per site 2019  Median [IQR] | | Total per site 2020  Median [IQR] | | p-value |  |
| --- | --- | --- | --- | --- | --- | --- | --- | --- | --- | --- | --- | --- |
| A. All activities | | | | | | | | | | | |  |
| Full cohort | | | 300019 | | 263380 | | 223 [51, 647] | | 183.5 [55, 597.5] | | 0,36 |  |
| Adult Patients | | | 175431 | | 143654 | | 101 [22, 383.5] | | 87 [18, 298.5] | | 0,14 |  |
| Child Patients | | | 122019 | | 116258 | | 65 [13, 305] | | 54 [13, 275] | | 0,68 |  |
| Lockdown Periods^*^ | | | 85995 | | 33987 | | 64 [15, 189.25] | | 23.5 [8, 82.75] | | **<10^-4^** |  |
| Not Lockdown Periods | | | 214024 | | 229393 | | 157 [35.25, 464.5] | | 156.5 [47.25, 527] | | 0,28 |  |
| Different periods of the year | Winter | | 65041 | | 68875 | | 45.5 [10, 138.75] | | 47 [12.25, 154.5] | | 0,14 |  |
|  | First Lockdown^*^ | | 46019 | | 25444 | | 33 [8, 105.25] | | 19 [6, 59] | | **<10^-4^** |  |
|  | Deconfinement | | 49399 | | 44192 | | 37.5 [9, 112.75] | | 34 [10, 119] | | 0,76 |  |
|  | Summer break | | 36973 | | 32930 | | 28 [7, 91] | | 24 [7, 80.5] | | 0,39 |  |
|  | Fall | | 53170 | | 47733 | | 39.5 [10, 123] | | 38 [11, 116] | | 0,94 |  |
|  | Second Lockdown | | 39942 | | 35663 | | 31 [8, 93] | | 33 [9, 95.75] | | 0,76 |  |
|  | Christmas break | | 9475 | | 8543 | | 9 [3, 24] | | 9 [3, 27] | | 0,5 |  |
| B. All face-to-face activities | | | | | | | | | | | |  |
| Full cohort | | | 292634 | | 235534 | | 217.5 [51, 638.75] | | 170 [50, 545.25] | | **0,088** |  |
| Adult Patients | | | 172237 | | 127842 | | 100.5 [22, 384.5] | | 72 [16, 284.5] | | **0,027** |  |
| Child Patients | | | 118015 | | 104618 | | 64 [13, 304] | | 54 [12.75, 259] | | 0,47 |  |
| Lockdown Periods | | | 84106 | | 21960 | | 64 [15, 179.75] | | 16.5 [6, 51.5] | | **<10^-4^** |  |
| Not Lockdown Periods | | | 208528 | | 213574 | | 155 [35.25, 459.75] | | 153.5 [45, 481] | | 0,51 |  |
| Different periods of the year | Winter | | 63298 | | 66963 | | 45 [10, 138.75] | | 47 [12, 145.75] | | 0,15 |  |
|  | First Lockdown^*^ | | 44854 | | 13917 | | 33 [8, 103.25] | | 10 [4, 31.25] | | **<10^-4^** |  |
|  | Deconfinement | | 48146 | | 37257 | | 37.5 [9, 109.25] | | 30 [8.5, 93.5] | | **0,096** |  |
|  | Summer break | | 35964 | | 31093 | | 28 [7, 89] | | 24 [7, 75.25] | | 0,3 |  |
|  | Fall | | 51906 | | 45225 | | 39.5 [10, 123] | | 37 [10.25, 110] | | 0,79 |  |
|  | Second Lockdown | | 39222 | | 33036 | | 30 [8, 90.5] | | 30 [8.75, 89] | | 0,79 |  |
|  | Christmas break | | 9244 | | 8043 | | 9 [3, 24] | | 9 [3, 25] | | 0,79 |  |
| C. Telehealth activities | | | | | | | | | | | |  |
| Full cohort | | | 7385 | | 27846 | | 2 [1, 14] | | 19 [4, 72.25] | | **<10^-4^** |  |
| Adult Patients | | | 3194 | | 15812 | | 2 [1, 11.25] | | 12 [3, 43] | | **<10^-4^** |  |
| Child Patients | | | 4004 | | 11640 | | 2 [1, 14.25] | | 9 [2, 44.5] | | **<10^-4^** |  |
| Lockdown Periods | | | 1889 | | 12027 | | 2 [1, 13] | | 13 [3, 39] | | **<10^-4^** |  |
| Not Lockdown Periods | | | 5496 | | 15819 | | 2 [1, 14.25] | | 11 [3, 44.5] | | **<10^-4^** |  |
| Different periods of the year | Winter | 1743 | | 1912 | | 3 [1, 16] | | 2 [1, 7] | | 0,57 | | |
|  | First Lockdown | 1165 | | 11527 | | 3 [1, 12] | | 13 [3, 38.75] | | **<10^-4^** | | |
|  | Deconfinement | 1253 | | 6935 | | 2 [1, 16] | | 7 [3, 26] | | **0,0023** | | |
|  | Summer break | 1009 | | 1837 | | 3.5 [1, 25] | | 3 [2, 10] | | 0,53 | | |
|  | Fall | 1264 | | 2508 | | 3 [1, 28.5] | | 4 [2, 11] | | 0,83 | | |
|  | Second Lockdown | 720 | | 2627 | | 2 [1, 14.5] | | 4 [2, 14] | | **0,027** | | |
|  | Christmas break | 231 | | 500 | | 1 [1, 6] | | 2 [1, 5] | | 0,64 | | |

**Supplementary table 1: Wilcoxon Tests to compare 2019 and 2020 activities in the BNDMR cohort.**

The font of p-values below 0.05 have been increased in size and bolded while p-values between 0.1 and 0.05 considered as trends have been bolded

|  | Total 2019  N | Total 2020  N | Total per site 2019  Median [IQR] | Total per site 2020  Median [IQR] | p-value |
| --- | --- | --- | --- | --- | --- |
| Consultation^*^ | 194328 | 154348 | 143 [32, 417.25] | 115 [28.5, 319] | **0,066** |
| Outpatient Hospitalization | 48217 | 38520 | 23 [5, 90.75] | 17.5 [4, 71.25] | 0,14 |
| Inpatient Hospitalization | 25447 | 19498 | 16 [4, 65] | 14 [4, 47] | 0,19 |
| Pluridisciplinary consultations | 24642 | 23168 | 16 [3, 74] | 14.5 [3, 68.25] | 0,76 |
| Telehealth activities^*^ | 7385 | 27846 | 2 [1, 14] | 19 [4, 72.25] | **<10^-4^** |

**Supplementary Table 2: Wilcoxon Tests to compare 2019 and 2020 activities by context in the BNDMR cohort**

The font of P-values below 0.05 have been increased in size and bolded while p-values between 0.1 and 0.05 considered as trends have been bolded

| Network name | Description | 2019 | 2020 | |
| --- | --- | --- | --- | --- |
| AnDDI | Developmental abnormalities, malformations and intellectual disability | 31575 | 30971 | |
| BRAIN TEAM | Central nervous system diseases | 6976 | 6695 | |
| Cardiogen | Hereditary cardiac diseases | 8745 | 8468 | |
| DefiScience | Rare diseases affecting Cognitive development and causing intellectual disability | 14712 | 15089 | |
| FAIIR | Autoimmune and autoinflammatory diseases | 20819 | 20638 | |
| FAVA-Multi | Marfan Syndrome | 4944 | 4527 | |
| FILFOIE | Rare liver diseaes | 9399 | 9387 | |
| FILNEMUS | Neuromuscular diseases | 38920 | 30683 | |
| FILSLAN | Amyotrophic lateral sclerosis | 9650 | 8684 | |
| FIMARAD | Skin diseases | 19642 | 15721 | |
| FIMATHO | Rare thoracic diseases | 3902 | 3111 | |
| FIRENDO | Endocrinology | 24085 | 16245 | |
| G2M | Metabolic diseases | 14874 | 13738 | |
| MARIH | Immuno-hemateologic diseases | 10767 | 8389 | |
| MCGRE | Rare red blood cells diseases | 6035 | 6948 | |
| MHEMO | Constitutional haemorrhagic diseases | 4880 | 5787 | |
| MUCO | Cystic fibrosis | 578 | 569 | |
| NEUROSPHINX | Rare pelvic and spinal cord malformations | 1647 | 560 | |
| ORKID | Kidney Diseases | 32530 | 28624 | |
| OSCAR | Bones, Calcium and cartilage diseases | 6461 | 6049 | |
| RESPIFIL | Respiratory diseases | 2377 | 2161 | |
| SENSGENE | Rare sensory diseases | 9319 | 7564 | |
| TETE COU | Head, Teeth and Neck diseases | 17182 | | 12772 |

**Supplementary table 3: Numbers of activities by specialty network in France**
